# Efficacy of immersive extended reality (XR) interventions on different symptom domains of schizophrenia spectrum disorders. A systematic review

**DOI:** 10.3389/fpsyt.2023.1208287

**Published:** 2023-08-04

**Authors:** Roope Holopainen, Jari Tiihonen, Markku Lähteenvuo

**Affiliations:** ^1^Department of Forensic Psychiatry, Niuvanniemi Hospital, University of Eastern Finland, Kuopio, Finland; ^2^Department of Clinical Neuroscience, Karolinska Institutet, Stockholm, Sweden; ^3^Center for Psychiatry Research, Stockholm City Council, Stockholm, Sweden

**Keywords:** schizophrenia, psychotic disorder, extended reality, virtual reality, augmented reality, treatment, rehabilitation, review

## Abstract

**Introduction:**

Extended reality (XR) is an umbrella term for virtual reality (VR) and augmented reality (AR), both novel vectors for therapeutic intervention modalities. In VR, head-mounted devices (HMD) allow interaction with three-dimensional virtual environments and simulated avatars, while AR overlaps virtual, simulated objects to observe physical reality. Treatment through immersive VR has been studied in psychiatry, including patients suffering from schizophrenia spectrum disorders, while there has not been much attention to AR technologies in psychiatry. Our systematic review aimed to examine the currently available literature regarding the treatment efficacy of immersive VR or AR technologies on different symptom domains of schizophrenia spectrum disorders, screen for potential adverse effects, and gather data on the technological and human resource requirements of such interventions to help guide future research.

**Methods:**

We conducted a systematic literature review with database searches carried out between 9/2021 and 8/2022 through PubMed, Scopus, EBSCOhost Academic Search Premier, and Web of Science.

**Results:**

We identified 2,157 records, 214 were assessed further for eligibility and 12 met inclusion criteria. All included articles studied immersive VR and none used AR technology. Included studies were heterogenous in nature, including AVATAR therapy (3) and CBT-based (5) VR interventions, as well as cognitive (2), social (1), and relaxation (1) training through VR. The comparison groups were either passive controls (waitlist and treatment as usual), therapeutic interventions (CBT and Integrated psychological treatment), passive VR environments, or traditional, comparable, non-virtual treatment modalities (social roleplay and progressive muscle relaxation training). Pooled together, the included studies on VR show positive treatment effects in all major symptom domains of schizophrenia spectrum disorders with hardly any adverse effects related to the intervention modalities.

**Conclusions:**

In this review, we have showcased how different symptom domains can be targeted through VR interventions, highlighting VR as a potential new vector for a diverse range of psychosocial therapeutic modalities that allow for completely new possibilities in the treatment of schizophrenia spectrum disorders. VR technology still requires more research and validation. Our review also shows that there are currently no studies examining AR technology in the treatment of schizophrenia spectrum disorders, indicating a distinctive research gap.

## 1. Introduction

Schizophrenia is a syndrome characterized by an assortment of heterogenous and diverse symptoms, with the core features usually divided into positive, negative, and cognitive categories (1). Positive (or psychotic) symptoms manifest as a loss of contact with reality, disorganization of thought, delusions, and hallucinations. The negative symptoms include impaired motivation, anhedonia, affective flattening, reduction of spontaneous speech, and social withdrawal while the cognitive symptoms manifest as a wide array of cognitive impairments. The positive symptoms tend to relapse and remit, while the negative and cognitive symptomology often causes severe and chronic, long-term functional impairment (1, 2).

Schizophrenia and its treatment impact the global economy widely, with estimations of the total economic burden of the patient population reaching an estimated range of 0.02 to 1.65% of the gross domestic product at national levels according to a systematic review (3). Most of the total costs (50 to 85%) were associated with indirect costs, which could indicate that the treatments and services as of now are insufficient to treat the full facet of the problems faced by the patient population (3). With refractory symptomology and associated comorbidity, treatment resistance, and reduced quality of life and life expectancy, as well as high disability levels in the patient group (1–4), the psychiatric field desperately requires new effective and easily deliverable treatment modalities for patients with schizophrenia to augment those already available.

Extended reality (XR) is an umbrella term, including technological solutions such as virtual reality (VR), augmented reality (AR), and mixed reality (MR), a technology utilizing aspects of both through environments in which the real world together with virtual objects and stimuli are presented together within a single percept (5–7). Multiple definitions for this continuum of virtuality and reality exist within the field (5–7). These modern tools are used to virtually generate environments or objects and allow for the creation of virtual scenarios that would be impossible or impractical to recreate in physical reality. The effortlessness of visualization and immersion into the virtually generated world can be utilized in a variety of ways to administer or augment therapeutic approaches while simultaneously allowing for the real-time observation, easy repetition, and scoring of such situations and protocols and their efficacy (5–7).

Fully or partially virtual worlds can be constructed in different ways. At present, four basic “reality” categories exist in the field: (1) physical reality or the real world; (2) augmented reality, where computer-based imagery is superimposed on the real-world image; (3) augmented virtuality, where real-life data are superimposed on the computer-generated world; and (4) VR, where the world is entirely computer-generated (6). Mixed reality, as priorly explained, mixes multiple categories (5).

Studies support the potential of VR technologies in their usability to treat psychiatric disorders ranging from, e.g., neurodevelopmental issues to psychoses to depressive and anxiety disorders (8), as well as an objective measurement tool in psychiatric diagnostics (9). The lucrative possibilities that real-time observation of the patient's psychological coping mechanisms in ecologically valid situations offer for the psychiatric research field have also been noted (10). VR technology can even be self-guided and ambulatory, possibly allowing for self-treatment at home (11). As a new tool, the evidence on VR technology is still somewhat preliminary and requires further validation.

In medical literature, there is a lot of ambiguity about the term “virtual reality,” which is often confused or used interchangeably with computerized approaches utilizing screens and gamified treatment modalities such as serious or exergaming (12), the former meaning the use of games in treatment and the latter exercising via game-like systems. Some prior reviews (13, 14) have differentiated immersive VR from such approaches. Immersive VR uses Head-Mounted Devices (HMD) to visually transport the patient to a different, virtually simulated three-dimensional environment, isolating them visually from physical reality, meaningfully improving immersion. Usually, the patient can interact with the virtual environment through a controller and movement.

AR can be used through a larger variety of media; through smartphone and tablet cameras or specifically created headwear that enhances the physical reality with superimposed imagery (15). There is scarce research on AR in the treatment of psychiatric disorders with some explorative studies on neuropsychiatric disorders and phobias (16). AR has mostly been studied in surgical fields or medical education (6).

Prior reviews have examined the effects of immersive and non-immersive VR interventions and examined the validity of VR technology as an assessment and treatment tool for neuropsychiatric, psychotic, and schizophrenia spectrum disorders or with paranoia and cognition as targets for intervention or evaluation (13, 14, 17, 18).

To expand and update on these prior reviews, we carried out a systematic review to study the available evidence on the treatment efficacy of immersive VR and AR technologies in the treatment of different symptom domains of schizophrenia spectrum disorders.

To complement prior reviews and to better clarify the effects of the interventions in the studied population, we chose to include only articles studying the treatment effects of immersive XR technologies (both VR and AR, as a clear distinction between the two might not always be evident), focusing only on patients suffering from schizophrenia spectrum disorders. For these goals, we chose to exclude studies utilizing non-immersive screen-based technologies for treatment and XR-based methods for the assessment of symptoms. We also excluded studies of healthy populations (e.g., trait paranoia or those in ultra-high risk for psychoses) and studies including populations with etiologically clearly different causes for psychoses (such as mood disorder-based psychoses). We aimed to examine the reported effects for all major symptom categories of schizophrenia spectrum disorders (namely positive, negative, and cognitive symptoms), including social and comorbid symptomology as categories as well.

To control the quality of the studies, only peer-reviewed studies with clear comparison groups were chosen to be included and a risk-of-bias assessment was carried out. Furthermore, to better inform future research and clinical work for individualized treatment and study protocols, we also screened the studies for possible adverse events and gathered available data on the technological and human resources requirements of included studies.

## 2. Methods

We performed a systematic literature search using PubMed, EBSCOhost Academic Search Premier, SCOPUS, and Web of Science databases on 28 September 2021.

The search words were chosen so we could identify all relevant studies targeting the population, interventions, and intervention targets of interest. Words near in meaning were also used to make sure no important studies would be missed. The following search words and their permutations were used with Boolean logic operators as each database utilized a slightly different search engine:

(Schizophrenia, “schizophrenia spectrum disorder,” “psychotic disorder,” psychosis) AND (“Virtual Reality,” VR, “Virtual Reality Exposure Therapy,” “Virtual Reality Therapy,” “Augmented Reality,” AR, “Extended reality,” XR, “Mixed reality,” MR, “Augmented Virtuality,” “Avatar therapy”) AND (rehabilitation, training, application, game, intervention, therapy, treatment) AND (negative, cognitive, “negative symptom,” “cognitive symptom,” social, refractory, motivation, function, impairment, affect, anhedonia, withdrawal).

Depending on the possibilities of each database, searches were restricted to clinical trials and randomized controlled trials, and articles in peer-reviewed medical journals written in English language. All databases were searched using search fields for keyword, title, and abstract information. For PubMed, appropriate MeSH terms were also used.

After the literature search, article lists from each database were imported into Mendeley reference management software (Ver 1.19.8., Mendeley Ltd). In Mendeley, we ran a check for duplicates, which were then excluded. Then, we screened the articles based on article title and abstract until only relevant articles were left for further review.

To update the search results, a complementary database search was conducted with the same search parameters on 22 August 2022.

The relevant articles were manually read to decide whether they were to be included in the review. In case of uncertainty, the article was discussed between R.H. and M.L. (Roope Holopainen and Markku Lähteenvuo, respectively) until a decision to include or not to include the article in the review was reached.

Articles were included based on the PICO (Patient, Intervention, Comparison, Outcome) protocol (19).

The inclusion criteria were as follows:

Original clinical trials and randomized controlled trials reported in English.

1. Studies targeting patients suffering from schizophrenia spectrum disorders.
2. Studies using immersive virtual reality, augmented reality, or similar immersive technology for intervention. Defined as the use of a head-mounted device for immersion.
3. Studies including a comparison/control group.
4. Studies including outcome measures targeting refractory positive, negative, cognitive, or social domain symptoms or comorbid psychiatric symptomology.

To assess the risk of bias in the studies included, we used the Cochrane risk-of-bias tool ROB 2, meant to assess randomized trials (20), and the ROBINS-I tool, meant for assessing non-randomized studies of interventions (21). The risk assessments were carried out on primary outcomes measuring targeted symptoms.

## 3. Results

The systematic literature search yielded 2,247 results. After removing duplicates, 1,848 results remained. After exclusion based on title and abstract, 176 articles were selected for further review.

After conducting a complementary search, 384 articles were identified, and after the removal of duplicates, 309 remained; 38 articles were selected for further review after exclusion based on name and abstract.

In total, 214 articles were analyzed, and 202 articles were excluded. The reasons for exclusion were as follows: article not in English (*n* = 10), review, theoretical paper, brief or a conference paper (*n* = 36), case study (*n* = 5), study protocol paper (*n* = 13), the article did not study an intervention with an effect on the studied outcome measures (*n* = 85), used methods were not immersive VR or AR technology (*n* = 32), did not study patients with a schizophrenia spectrum disorder (*n* = 8), the article did not have a comparison group (*n* = 3), and not the original study population or a sub-cohort (*n* = 10).

Three of the exclusions were discussed between RH and ML and were excluded (1) because the immersivity of VR could not be found out in the article and (2) because of mixed patient populations.

**Figure 1:**
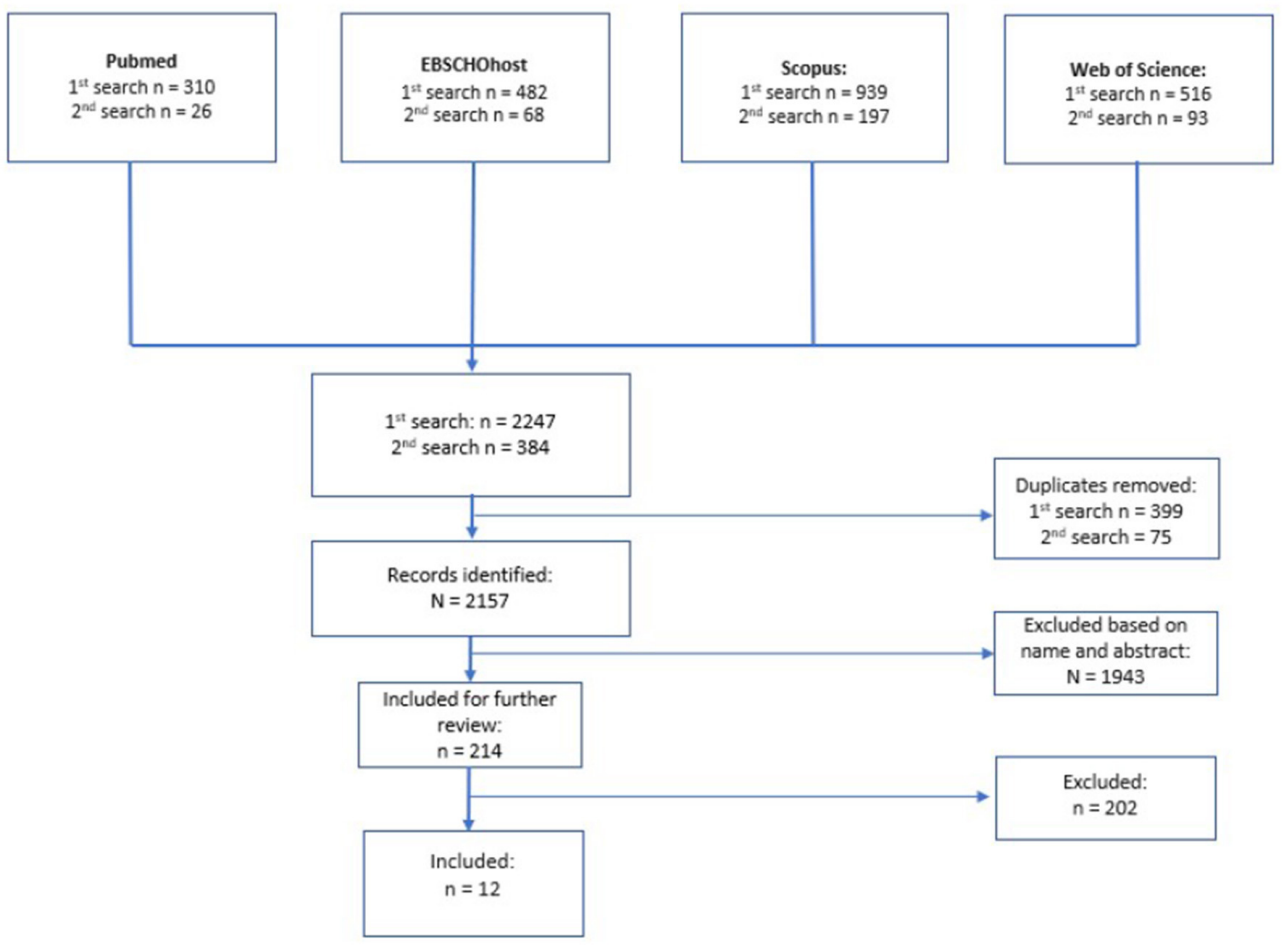
Modified PRISMA flowchart of the systematic literature search.

After careful evaluation of each article, 12 articles were included in the review. For the full flowchart of the systematic search, see the PRISMA flowchart (22) in Figure 1.

## 4. Intervention types

### 4.1. Augmented reality interventions

We could not identify any study utilizing immersive AR technology in the treatment of patients with schizophrenia.

### 4.2. Virtual reality interventions

**Figure 2:**
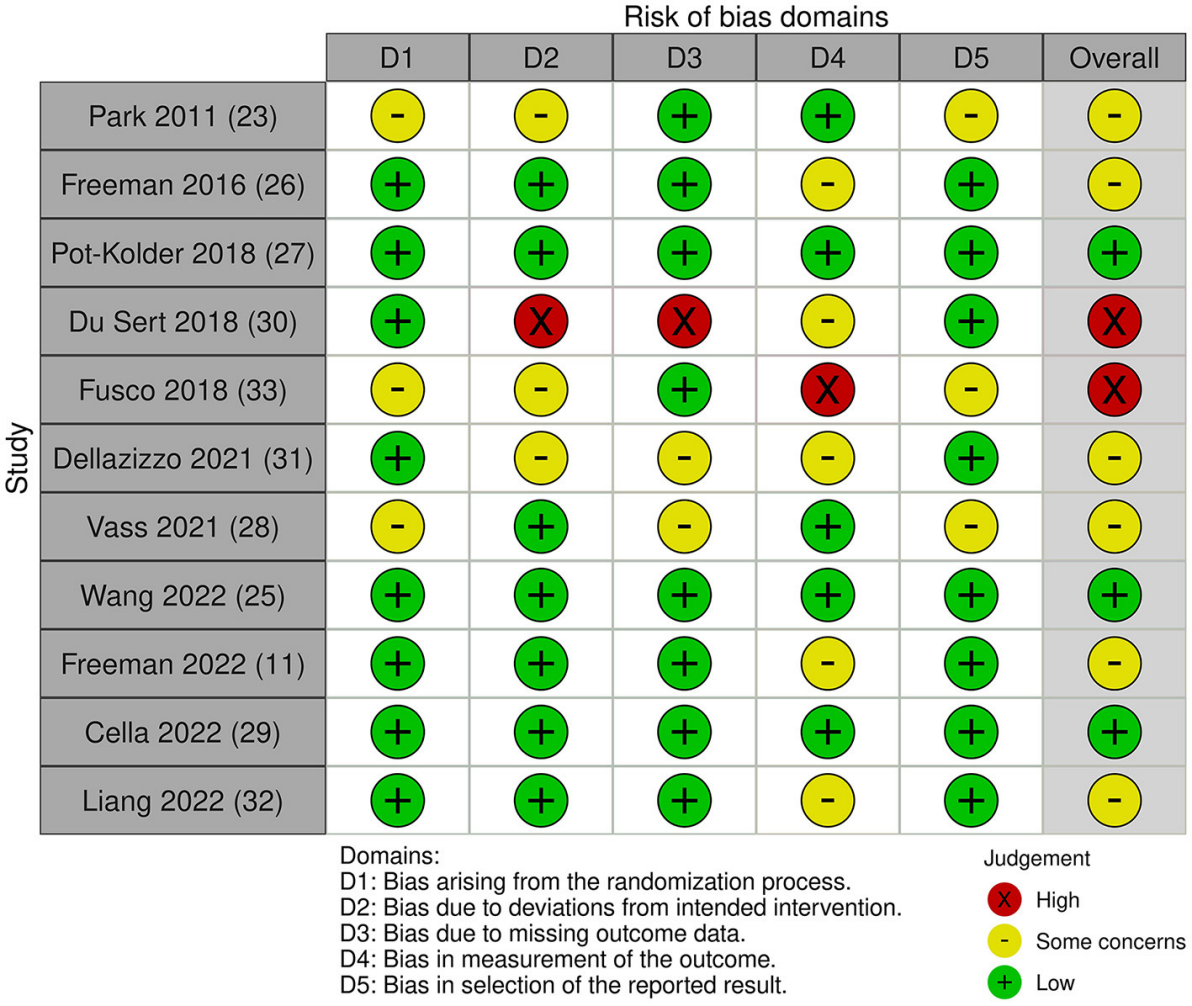
Risk-of-bias assessment for included studies (randomized controlled trials).

**Figure 3:**
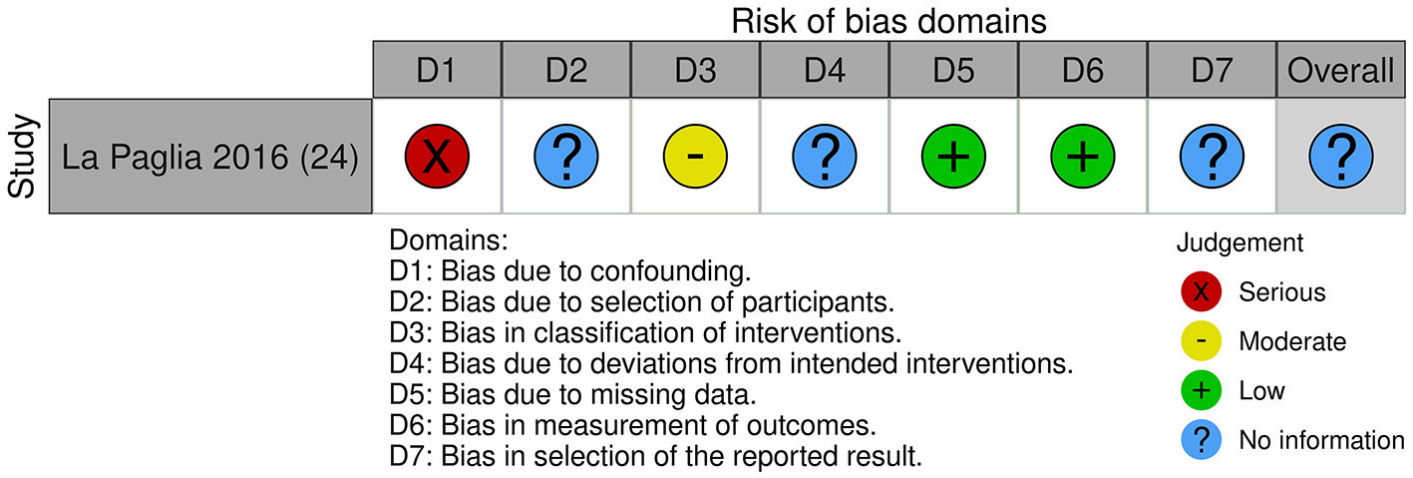
Risk-of-bias assessment for included studies (clinical trials).

We could broadly differentiate five intervention types utilizing immersive VR in this review: social training, cognitive training, cognitive behavioral therapy-based interventions, avatar therapy-based interventions, and relaxation training. The characteristics of different intervention modalities and research studies are listed in Tables 1, 2, respectively. The risk-of-bias assessments are presented in Figures 2, 3.

**Table 1:** Intervention characteristics.

**Intervention modality**	**Description**
VR Social training	Immersive virtual environments, scenarios, game avatars, and dialogue are used to help train the patient to be more socially fluent.
VR Cognitive training	Diverse virtual reality tasks are used to help patients rehabilitate certain aspects of their (neuro)cognition.
VR CBT-based intervention	Immersive virtual environments are used to augment cognitive and behavioral therapeutic methods, often with the goal of social exposure.
Avatar therapy-based intervention	Avatar therapy is a therapeutic method, which uses a virtual, audiovisual construction of the patient's audiovisual hallucination through therapist-guided roleplay to practice different responses. VRT and AVATAR therapy are differentiated by the use of HMD as the vector of intervention. CATS adds real-time full-body and facial motion capture to VRT to help the therapist animate the avatar.
VR Relaxation training	Virtual environment is used in the augmentation or creation of a relaxing effect.

VRT, Virtual reality-assisted therapy; CATS, virtual reality-based computer AT system.

**Table 2:** Study characteristics.

**Study**	**Year**	**Study design**	**Country**	**Diagnosis**	**Total sample size**	**Intervention type**	**Control condition**	**Primary Outcome measure**	**Secondary Outcome measures**	**Main finding**
Park et al. (23)	2011	RCT	South Korea	Schizophrenia	64/91	VR Social Training	Traditional social roleplay	Social skills: voice quality, nonverbal skills, conversational properties. Social competence: unstructured roleplay test (10 roleplay tests, SBS)	RAS, RCS, SPSI-R, Interest-in-Participation Questionnaire, and Right-or-wrong questions regarding the session.	Social skills training (SST) through virtual reality roleplay improved conversational skills and assertiveness more than SST through traditional roleplay, but nonverbal skills less.
La Paglia et al. (24)	2016	Clinical trial	Italy	Schizophrenia	15	VR Cognitive training	Integrated Psychological Treatment	MMSE, FAB, TMT (A, B, B-A), ToL, WCST	-	VR cognitive training improved neurocognition (general cognitive functioning, planning skills, and sustained attention) in comparison to integrated therapy.
Freeman et al. (25)	2016	RCT	UK	Schizophrenia, schizoaffective disorder, delusional disorder, or psychosis NAS	30	VR CBT-based intervention.	VR environment exposure	Conviction of paranoid beliefs, distress related to the beliefs, real-life social behavior test (VAS-scale ).	-	A brief immersive VR cognitive therapy (dropping of safety behaviors) led to a significant reduction in paranoid beliefs and distress in comparison to simple VR exposure.
Pot-Kolder et al. (26)	2018	RCT	Netherlands	Psychotic disorder	116	VR CBT-based intervention	Wait-list	ESM	SBQ, GPTS, BDI, SOFAS, MANSA, BCCS, BARS, IPQ, SSQ	Immersive VR CBT -therapy reduced paranoid ideation, momentary anxiety, and safety behaviors in real-life social situations, but did not significantly increase social participation. The results remained significant at follow-up.
Du Sert et al. (27)	2018	RCT	Canada	Schizophrenia or schizoaffective disorder.	19	Avatar therapy-based intervention	TAU	PSYRATS	BAVQ-R, Q-LES-Q-SF, BDI-II, PANSS	Avatar therapy in immersive virtual reality (VRT) showed a large therapeutic effect in treatment-resistant schizophrenics compared to TAU. The effects remained significant at 3-month follow-up.
Fusco et al. (28)	2018	RCT	Italy	Schizophrenia spectrum disorder or other psychotic disorder	22	VR relaxation training	PMR	BAI, STAI-Y	-	Progressive Muscle Relaxation (PMR) in immersive VR was more effective in reducing anxiety than traditional PMR.
Dellazizzo et al. (29)	2021	RCT	Canada	Schizophrenia or schizoaffective disorder.	74	Avatar therapy-based intervention	CBT	PSYRATS	BAVQ-R, Q-LES-Q-SF, BDI-II, PANSS	Avatar therapy in immersive virtual reality (VRT) found similar and even superior effects compared to CBT in treatment-resistant schizophrenics, with effects lasting at 12-month follow-up.
Vass et al. (30)	2021	RCT	Hungary	Schizophrenia	17/21	VR CBT-based intervention	Passive VR environment	PANSS, neurocognitive deficits (RBANS, WCST), BCMET, faux pas test, cartoon stories, Hungarian metaphor and irony test, LQoLP, SSQ, patient's subjective opinion of intervention, subjective evaluation of perceived changes by a relative of a patient.	-	A Theory of mind intervention through immersive virtual reality led to diverse effects in comparison to passive VR environment exposure in patients suffering from schizophrenia.
Wang et al. (31)	2022	RCT	China	Schizophrenia	64	Cognitive training	TAU	B-CATS (Including DSST, TMTA, and B, AF	-	Intensive, immersive, and active VR serious games in addition to standard psychiatric care can improve working memory and executive function in patients with schizophrenia.
Freeman et al. (11)	2022	RCT	UK	Schizophrenia Spectrum Disorder or an affective disorder with psychotic symptoms	346	VR CBT-Based intervention	TAU	O-AS	MIA, CSSR, RGPTS, PWQ, PHQ, O-BAT, EQ-5D, ReQoL, QPR. Activity levels are measured using actigraphy (over 7 days), and a time budget assessing meaningful activity (that considers the complexity of activities and effort required).	TAU augmented by automated immersive VR therapy led to significant reductions in anxious avoidance of, and distress in, everyday situations compared to usual care alone in psychosis patients. Especially in severe agoraphobia.
Cella et al. (32)	2022	RCT	UK	A documented episode of psychosis or a schizophrenia diagnosis.	30	VR CBT-Based intervention	TAU	GAS	CAINS, SNS, WSAS, EEfrT, WCST, semi-structured subjective feedback on the intervention.	TAU augmented by CBT delivered through immersive virtual reality was useful in supporting patients' recovery goals in comparison to TAU alone in a pilot RCT feasibility study.
Liang et al. (33)	2022	RCT	China	Schizophrenia	65	Avatar therapy-based intervention	CBT	PSYRATS	P300 recording, BAVQ-R, PANSS, HAMD, HAMA, SES, Q-LES-Q-SF.	Avatar Therapy-based intervention through immersive VR (CATS) was similar in treatment effect of AVH in comparison to CBT in patients with treatment-resistant schizophrenia. The changes in PSYRATS and BAVQ-R scores correlated with changes in P300 amplitudes.

Abbreviations for outcome measures: SBS, Trower's Social Behavior Scales; RAS, Rathus Assertiveness Schedule; RCS, Relationship Change Scale; SPSI-R, Social Problem Solving Inventory -Revised; MMSE, Mini-Mental State Examination; FAB, Frontal Assesment Battery; TMT A; B; B-A, Trail Making test (forms A,B and B-A; respectively); ToL, Tower of London; WCST, Wisconsin Card Sorting Test; VAS, Visual Analoq Scale; ESM, Experience Sampling Method; SBQ, Safety Behavior Questionnaire; GPTS, Green Paranoid thoughts Scale; SIAS, Social Interaction Anxiety Scale; BDI, Beck Depression Inventory; SOFAS, Social and Occupational Functioning Assessment Scale; MANSA, Manchester Short Assessment of Quality of Life; ISMI, Internalized Stigma of Mental Illness questionnaire; BCCS, Brief Core Schema Scale; DACOBS, the self-reported Davos Assessment of Cognitive Biases Scale; BARS, Brief Adherence Rating Scale; IPQ, Igroup Presence Questionnaire; SSQ, Simulator Sickness Questionnaire; PSYRATS, Psychotic Symptoms Rating Scale; BAVQ-R, Beliefs About Voices Questionnaire-Revised; Q-LES-QF, Quality of Life Enjoyment and Satisfaction Questionnaire-Short Form; BDI-II, Beck Depression Inventory-II; PANSS, Positive and Negative Symptoms Scale; BAI, Beck Anxiety Inventory; STAI-Y, State Trait Anxiety inventory form Y; RBANS, Repeated Battery for the Assessment of Neuropsychological Status; BCMET, Baron-Cohen Mind in the Eyes Test; LQoLP, Lancashire Quality of Life Profile; B-CATS, Brief Cognitive Assessment Tool for Schizophrenia; DSST, Digital Symbol Substitution Test; AF, Animal Fluency; O-AS, Oxford Agoraphobic Avoidance Scale; MIA, Agoraphobia Mobility Inventory-Avoidance scale; CSSR, Columbia Suicide Severity Rating Scale; RGPTS, Revised Green et al. Paranoid Thoughts Scale; PWQ, Paranoia Worries Questionnaire; PHQ9, Patient Health Questionnaire; O-BAT: Oxford-Behavioral Assessment Task; EQ-5d, Quality of life - five-level EQ-5D; ReQoL, Recovering Quality of Life questionnaire; QPR, Questionnaire about the Process of Recovery; GAS, The Goal Attainment Scaling; CAINS, The Clinical Assessment Interview for Negative Symptoms; SNS, The Self-evaluation of Negative Symptoms; WSAS, The work and social adjustment scale; EEfrT, Effort Expenditure for Reward Task; HAMD, Hamilton Depression Scale; HAMA, Hamilton Anxiety Scale; SES, Rosenberg self-esteem scale. Abbreviations for treatments: VR, Virtual Reality; PMR, Progressive muscle relaxation; CBT, Cognitive Behavioral Therapy; TAU, Treatment As Usual; VRT, Virtual Reality Assisted Therapy; CATS, virtual reality–based Computer Avatar Therapy System . Other abbreviations: AVH, Audiovisual hallucination; ESM, Experience Sampling Method; SBQ, Safety Behavior Questionnaire; GPTS, Green Paranoid.

One study utilized VR social training (23), two VR cognitive training (24, 31), five VR-CBT-based intervention methods (11, 25, 26, 30, 32), three avatar therapy (27, 29, 33), and one relaxation training (28).

## 5. Rehabilitation of symptom domains

### 5.1. Refractory positive symptoms

#### 5.1.1. Avatar therapy interventions

Du Sert et al. (27) carried out a pilot study for a randomized, partial cross-over clinical trial for avatar therapy-based VR-assisted therapy in comparison to treatment-as-usual (TAU). The patients suffered from treatment-resistant schizophrenia (defined as not responding to at least two antipsychotic medications), with half of the final sample unresponsive even to clozapine. Also, 19 patients (with 15 finishing the treatment and follow-up) were randomly assigned in a 1:1 manner to either 7 weeks of VR-assisted (avatar) therapy (VRT) or control condition (TAU) without blinding. After the first treatment period, the TAU group received the VRT as well. Follow-up was scheduled 3 months after the last VRT therapy session.

The outcome measures for the study were changes in psychotic symptoms (Psychotic Symptoms Rating Scale, PSYRATS) and beliefs related to audiovisual hallucinations (AVH) (Beliefs About Voices Questionnaire-Revised, BAVQ-R), positive and negative symptoms (PANSS), depression symptomology (BDI-II), and quality of life were also assessed (Quality of Life Enjoyment and Satisfaction Questionnaire-Short Form). The presence of persecutory AVH, fear, and anxiety after each session were also measured on a 0–10 numerical scale.

Patients in the experimental group received weekly six 45-min sessions of avatar therapy and before this, one meeting was used to create a personified, virtual avatar of their persecutory AVH (most distressing or dominant in case of multiple AVHs) which would be controlled by the therapist in subsequent sessions. The sessions included confronting the avatar to improve emotional regulation and assertiveness and gain self-esteem, as well as consolidation sessions to apply previously learned skills in the scenario. Throughout VRT, the avatar's interactions grew less abusive and more supportive. Three patients received 1 to 4 additional consolidation sessions.

The psychiatric symptoms remained unchanged throughout the TAU period while there was a significant reduction in AVH symptoms seen (*p* < 0.01), especially regarding distress (*p* < 0.001), omnipotence, and malevolence related to the AVH (*p* < 0.05) during VRT. General psychiatric symptoms measured by PANSS and BDI were also reduced significantly (*p* < 0.05) and an improvement in quality of life was seen (*p* < 0.05). Improvements remained significant at the 3-month follow-up. There was no significant correlation between the number of therapy sessions and clinical outcomes. Patients also reported significant subjective decreases in anxiety and fear beginning at week 4 as the first 2 weeks of the intervention were seen as most anxiogenic and led to dropouts after the first sessions.

Dellazizzo et al. (29) expanded on the du Sert study with a 1-year-long RCT comparing VR-assisted therapy in adjunction to TAU (*n* = 37) to cognitive behavioral therapy in adjunction to TAU (*n* = 37) in patients with treatment-resistant schizophrenia. The intervention comprised 7–9 sessions administered weekly.

The study found a significant reduction in AVH symptoms between baseline and 3-month follow-up in both groups assessed by PSYRATS-AH with large effect sizes for VRT and small-moderate for CBT (*p* < 0.001, *d* = 1.080 for VRT; *p* = 0.001, *d* = 0.555 for CBT), most prominently on the frequency of and distress related to AVH (*d* = 0.701 and *d* = 0.998, respectively). VRT also showed significant improvement regarding persecutory beliefs, whereas the CBT findings were on trend level and not statistically significant. Both interventions showed moderate effects on persecutory beliefs about voices. Depressive symptoms diminished in both treatment groups and overall psychiatric general symptoms as measured with the PANSS significantly diminished with VRT (*p* = 0.008), while not significantly in the CBT arm. Most effects were observed on the excited/hostility and anxio-depressive subscales (*p* < 0.001). The effect of VRT was of moderate range for overall symptomatology and was found to be larger for affective symptoms. In addition, VRT significantly ameliorated quality of life with an effect of moderate magnitude.

Results for VRT were maintained long term up to the 1-year follow-up with no statistically significant differences from the 3-month follow-up for most outcomes, except for the engagement subscales of the BAVQ-R for VRT, which was found to diminish significantly (*p* = 0.002) from 3- to 12-month follow-up and returned to baseline. CBT showed no statistically significant differences in any of the outcomes.

Liang et al. (33) carried out a pilot RCT where they compared a 7–9-session VR-based computerized avatar therapy system (CATS) treatment to CBT in patients with treatment-resistant schizophrenia. Both groups continued to receive their normal psychopharmacologic treatment during the study, with no changes to dosage. The study recruited 65/85 eligible patients with 30/32 in the experimental group and 28/33 in the control group finishing the treatment and final assessments.

The intervention included 1 avatar creation session and six 60-min therapeutic sessions divided into three parts: (1) predialogue (review, discussion of objectives, and agree to focus on patient–avatar dialogue); (2) trialogue of 10–15 min among therapist (in a separate room), patient, and avatar of the AVH; and (3) postdialogue focusing on the feedback following the trialogue. The dialogue with the avatar was provided to the patient in MP3 format for continued use at home. The study measured hallucinations (PSYRATS), beliefs regarding hallucinations (BAVQ-R), psychiatric symptomology (PANSS, Hamilton Depression and Anxiety Scales, HAMD HAMA), self-esteem (Rosenberg self-esteem scale, SES), and quality of life before and after interventions and at 12-week follow-up. The study also measured electroencephalographic (EEG) data for visual P300 recording at baseline and post-interventions. The visual P300 component is an attention-dependent event-related potential (ERP), a neurological marker considered to reflect complex cognitive functions, such as selective attention and working memory.

From baseline to 12-week follow-up, the severity of AVH as measured by PSYRATS decreased significantly over time for both treatment groups (*p* < 0.001 for CATS and *p* = 0.012 for CBT) showing a reduction in distress related to (*p* < 0.001 for CATS and *p* = 0.020 for CBT) and frequency of AVH (*p* = 0.002 for CATS and *p* = 0.032 for CBT), with a large effect size for CATS and moderate for CBT (effect on AVH symptoms for VRT *d* = 1.230 and CBT *d* = 0.713). The CATS also significantly reduced beliefs of omnipotence regarding the AVH, while the effect for CBT was trend-level and not statistically significant. Improvement in positive symptoms for PANSS, self-esteem, and quality of life was only seen for the CATS group. For affective symptoms, the study did not find an interaction effect, but a significant time effect with the CATS group showing improvement in HAMA scores (*p* = 0.022). The study found significantly higher P300 amplitudes in the CATS group in comparison to the CBT group in post-intervention measures. The P300 amplitude change correlated with changes in PSYRATS total scores and BAVQR-O in the CATS group. No correlations were found for the CBT group.

#### 5.1.2. CBT-based interventions

Freeman et al. studied the effects of cognitive therapy tools in brief immersive VR therapy sessions in comparison to a simple VR exposure (in the same environment) in a small non-blinded RCT (25). The included 30 patients suffering from persecutory delusions with a diagnosis of schizophrenia, schizoaffective disorder, delusional disorder, or psychosis NAS were randomized either to the threat belief testing group (*n* = 15) or VR exposure group (*n* = 15).

For intervention, there were two VR environments that patients could move in: an underground train and a lift. The number of game avatars present varied depending on difficulty from 0 to 22 in the train and from 2 to 6 in the lift. Both groups used the same virtual environment but were given slightly different instructions at the start of immersion in the virtual environment. The first part of the instructions for both groups was the same, with a short explanation and encouragement for virtual exposure as a treatment tool. The last part of the instructions differed between the two groups; the exposure group was encouraged to use their safety behaviors while the therapy group was encouraged to drop them and test their threat beliefs in a VR environment. The whole intervention and testing took ~60–90 min with 30 min spent inside the VR environment.

Before the intervention, the patient's conviction in the delusions was rated, after which they completed a 5-min behavior testing in a real-life social environment of their choice and which they wanted help with. The tests were done either at a patient's home or at a hospital ward. The patients were then brought to a VR laboratory for the intervention, which included seven short VR periods and delusion conviction ratings before and after VR immersion. The key variables, the conviction of persecutory beliefs and distress, were assessed with visual analog scales at the end and beginning of the testing day, and before and after VR immersion. After the VR intervention, the behavior test and delusion ratings were repeated at home. The study also measured PANSS (positive subscale), PSYRATS (delusions), the Safety Behaviors Questionnaire (persecutory beliefs), BAI, and BDI before the testing.

The study found that the threat testing group was significantly more likely to move inside a social VR environment in comparison to the control group (additional 10.5 m, *p* < 0.001). There was no difference in the movement between the groups in a VR environment empty of people (virtual game avatars). For conviction in paranoid beliefs, a gradual and significant reduction for the threat testing group was seen throughout the VR scenarios while the scores remained stable in the control group. The conviction ratings by the final scenario were significantly more reduced in the threat belief testing group in measures of scores (additional 20.9% reduction, *p* < 0.001) taken before VR and in post-VR scores (additional 12.9% reduction, *p* = 0.039), with a mixed model of both variables showing additional reduction by 17.9% (*p* < 0.001). After the first scenario, there was no within-session (pre- to post-score) change in allocation to level, indicating that both variables are changing in parallel as the intervention went on. The same pattern of gradual reduction in the testing group was seen in distress related to paranoia, while the scores were stable in the exposure group. With the means of pre- and post-scores measured over the intervention, by the last scenario, there was a significantly higher reduction in the threat testing group in comparison to the control group (additional reduction of 17.6 points, *p* = 0.001). Overall, there was a significantly higher reduction (22%, *p* = 0.024, *d* = 1.3.) in the level of conviction to the paranoid beliefs in the testing group between the start and end of testing (79.8 to 46.5%) compared to the exposure group (78.5 to 67.6%).

For the repeated real-life social situation behavior test, the threat belief testing group found the task less distressing than the exposure group. After controlling for the level of distress caused by the real-world situation at the first time of entering, the brief VR cognitive therapy significantly reduced distress in the real-world situation in comparison to the exposure group (additional 19.6% reduction, *p* = 0.020, *d* = 0.8).

Pot-Kolder et al. (26) studied the effects of immersive virtual reality-based cognitive behavioral therapy in comparison to wait-list control on paranoid ideation and social participation in outpatients with psychotic disorder in a single-blinded RCT.

In total, 116 patients suffering from schizophrenia, schizoaffective disorder, delusional disorder or not-otherwise specified psychotic disorder, and simultaneous avoidant behavior and paranoid ideation were recruited from seven mental health centers. The participants were randomized in a 1:1 manner into a group receiving either VR-CBT treatment in addition to TAU (*n* = 58) or to wait-list control group (TAU, *n* = 58), who were then subsequently offered VR-CBT after a follow-up period. The physicians treating patients were asked to not change patient medication during the study period.

The intervention was CBT in an immersive VR environment (four locations: street, bus, cafe, and supermarket). Stimuli were controlled by the therapist and thus the experiences were personalized for each patient. The goal of the VR exercises was to guide exposure to cues in social environments that caused fear, paranoid ideation, and safety behaviors. The therapist communicated with the patients and guided them to explore and challenge thoughts and behaviors and to test harm expectancies in the VR environment. There was no homework or tasks given to patients between sessions. The VR-CBT was delivered in 16 sessions over 8 to 12 weeks, with each session lasting 60 min. The session included 40 min of social VR exercises and 20 min of planning and reflecting on exercises. Patients in both groups received treatment as usual, such as antipsychotic pharmacologic treatment. The control group had regular contact with psychiatrists and psychiatric nurses.

As the primary outcome, the study measured social participation; objectively (amount of time spent with others) and subjectively (momentary paranoia, perceived social threat, and momentary anxiety in company) through an Experience Sampling Method (ESM), a structured diary through a carried electronic device called PsyMate. It beeped at quasi-random time intervals 10 times per day for 6 days and asked patients to report the momentary thoughts, feelings, symptoms, social contexts, and appraisals of the contexts (7-point Likert scales). The patients had 15 min to answer, and for the measures to be included, at least one-third of the beeps had to be included (minimum 20 measurements). Time spent with others was measured by the proportion of beeps that participants reported to be in the company of others (excluding mental health professionals). Secondary outcomes for symptom measures were the Safety Behavior Questionnaire-Persecutory Delusions, Paranoid Thoughts Scale, Social Interaction Anxiety Scale, and Beck Depression Inventory, and for functional outcomes the Social and Occupational Functioning Assessment Scale and Manchester Short Assessment of Quality of Life. Stigma was assessed with the Internalized Stigma of Mental Illness questionnaire. To examine the putative working mechanisms of the therapy, cognitive constructs were assessed with the Brief Core Schema Scales and the self-reported Davos Assessment of Cognitive Biases Scale. Medication adherence was measured with the Brief Adherence Rating Scale. After the fourth and eighth sessions, presence in VR was assessed with the Igroup Presence Questionnaire and Cybersickness symptoms through the Simulator Sickness Questionnaire. The assessments were done at baseline, after the treatment period (3 months from baseline), and at follow-up (6 months after baseline). The analysis was intention-to-treat, and patients who dropped out were included.

In total, 11 patients in the VR-CBT group dropped out, 17 changes in psychiatric medication were reported, 96 participants completed the post-treatment assessment sufficiently, and 87 participants completed the follow-up. Both groups were similar at baseline, except for the use of safety behaviors, which was significantly lower in the control group.

The VR-CBT in comparison to TAU reduced momentary paranoid ideation (*p* < 0.0001, effect size = −1.49) and momentary anxiety (*p* = 0.0002, effect size = −0.75), with effect sizes remaining significant at follow-up. VR-CBT did not significantly improve social participation. Time spent with others decreased by 2.4% in the control group between baseline and the follow-up assessment, whereas the amount of time marginally increased by 0.3% in the VR-CBT group. No significant interaction effects were noted for perceived social threat at the post-treatment or follow-up assessments.

Compared with the control group, the use of safety behaviors decreased significantly in the VR-CBT group at both the post-treatment and follow-up assessment and at post-treatment (score of 28.8 in SBQ at baseline to 21.1 at post-treatment and 20.2 at follow-up). The largest reduction at the post-treatment visit was for the *in situ* safety behaviors subscale. Treatment effects of VR-CBT on paranoid ideation were significant in comparison to the control group: at the post-treatment and follow-up assessments regarding levels of ideas of persecution and social reference (Reduction in paranoid thoughts scale scores of 41.2 at baseline to 33.4 at post-treatment and 31.4 at follow-up for persecutory ideation and similarly from 43.6 to 35.4 and 34.0 for social reference). No significant change between groups was found for depression and anxiety or quality of life. The VR-CBT group had improvements in self-stigmatization and social functioning at follow-up, whereas the control group did not.

Part of the treatment effect for paranoid ideation at the follow-up was mediated by a change in safety behaviors and a change in social cognitive problems. Those who received VR-CBT used fewer safety behaviors and reported fewer social cognition problems than those in the control group, subsequently experiencing less paranoid ideation. The direct effect of the treatment on paranoid ideation was no longer significant after the inclusion of the mediators in the model. No significant mediators of VR-CBT were found for momentary paranoia. The total effect (independent of mediators) and direct effect (including the mediators) of treatment were both significant for momentary paranoia. Regarding the feeling of presence in the VR environments, participants reported sufficient presence.

### 5.2. Negative symptoms

Cella et al. (32) studied the effects of augmentation of normal psychiatric treatment (TAU, treatment as usual) of schizophrenia with immersive VR in comparison to TAU only for goal attainment, negative symptomology, and functioning.

In a single-blind, randomized, and controlled feasibility study, 29 out of 30 eligible patients with a documented episode of psychosis and/or schizophrenia diagnosis were randomized into either a treatment or control group (Experimental group, *n* = 14, TAU, *n* = 15).

The intervention studied was a 12-session virtual reality-negative symptom therapy (V-NeST) based on CBT and cognitive remediation techniques supported by virtual environments, where patients are encouraged to approach motivational and unique challenges ranging in motivational requirement (such as lounge for passive activities; music or watching tv, or a factory, game room, or a social space, where patients were asked to perform tasks or interact with avatars). The minimum number of intervention sessions to attend was six. The experiences were guided, supported, and discussed with a graduate-level psychotherapist with clinical experience with the target population. The therapist received tailored training and weekly supervision.

As the primary outcome, the study measured Goal attainment scaling (GAS), a scale reflecting the achievements of patients' pre-selected treatment goals. For secondary outcomes, Clinical assessment interview for negative symptoms (CAINS), self-evaluation of negative symptoms (SNS), Work and social adjustment scale (WSAS), and Effort expenditure for reward task (EEFrT) were used. The patients also completed semi-structured subjective feedback on the intervention. The study followed an intention-to-treat principle.

Although there was a higher level of adverse effects in the experimental group in comparison to TAU during the study (7 vs. 4), none of these were considered to be associated with the study participation. Therefore, 9 out of 15 patients in the experimental group were interviewed and only 6 out of 15 completed EEfRT and WCST tasks. The study observed a significantly large effect size in the primary outcome GAS in comparison to the TAU group (*p* = 0.001, *d* = 1.48) but the treatment effects observed for secondary outcomes (clinically and self-assessed negative symptomology) were considered by the authors to be too small and varied to draw any conclusion. The intervention was well received by the patients.

### 5.3. Social symptoms

Park et al. (23) compared social skills training through immersive VR to conventional roleplay methods in 91 in patients with schizophrenia. The patients were randomized into either virtual reality social skills training (VR-SST, *n* = 45) or social skills training through traditional roleplay (SST-TR, *n* = 46).

Both groups went through 10 semiweekly therapeutic sessions for 5 weeks, each lasting for 90 min. The manualized program included five sessions of conversational skill training, three sessions of assertiveness training, two sessions of emotional expression skills training, and included revision and homework. Each session included three roleplays with different scenes, first modeled by the therapist, then followed by the participant, after which feedback was given, and the scene was then repeated. The group delivering the interventions included a main therapist and co-therapists (social workers). The only difference between the two groups was in the materials used for the delivery of the intervention with the VR-SST group utilizing an immersive VR system and the SST-TR group verbal, writing, picture, and video material, as well as therapists as actors.

The primary outcomes studied were social skills and competence through unstructured roleplay tests, which were recorded and rated by a blinded evaluator using the SBS scale (SBS, Trower's Social BehaviorScale), e.g., voice, non-verbal, and conversational skills. Secondary outcomes were self-reports for assertiveness (RAS, Rathus Assertiveness Schedule), interpersonal relationship skills (RCS, Relationship Change Scale), and cognitive, affective, and behavioral responses to real-life problems (SPSI-R, Social Problem-Solving Inventory-Revised). The study also examined proxy methods for motivation (Interest-In-Participation Questionnaire) and generalization of skills (right-or-wrong questions related to the topics of corresponding sessions). Possible confounders that were controlled for were session duration, age, time spent for instructions, orientation, contact with the main therapist and therapist recommendations, group size of the intervention group (4 to 5), and protocols for encouraging attendance.

Both SSTs improved social skills overall, but there were differences between the interventions. VR-SST group showed better improvement on the SBS scale in conversational skill than SST-TR (F_1, 62_ = 17.261, *p* < 0.001, partial η^2^ = 0.218), but inferior improvement in non-verbal skills (F_1, 62_ = 6.201, *p* = 0.015, partial η^2^ = 0.091). The VR-SST intervention also showed greater improvement in assertiveness on the RAS (F_1, 62_ = 4.957, *p* = 0.030, partial η^2^ = 0.074). VR-SST group also scored higher in attendance than SST-TR (attendance rate 95.3 ± 6.8% and 91.0 ± 7.3%, respectively; t_62_ = 2.411, *p* = 0.0199), as well as on generalization of the learned skills.

Freeman et al. (11) studied the effects of an automated, immersive VR intervention in a multicenter, parallel-group single-blind RCT on patients suffering from schizophrenia spectrum disorder or an affective disorder with psychotic symptoms. In total, 174 patients were assigned to the intervention group and 172 to the control condition (TAU).

The intervention was an immersive, automated VR application “Gamechange,” which aimed to relearn safety by testing fear expectations through repeated behavioral self-experiment. Inside the VR environment, a programmed virtual coach helped to guide the therapy, modify and test behavior, and gave feedback. Participants selected one of six VR social scenarios (café, general practice waiting room, pub, bus, opening the front door of the home onto the street, and small local shop). Each scenario comprised five levels of difficulty (based on the number and proximity of people in the social situation and the degree of social interaction) and participants worked their way through each level. There were differences in the level of interaction with the avatars and the level of attention given to the participant by the avatars depending on difficulty. After finishing a scenario, the participant could choose a different scenario in each session or repeat a previous scenario and/or level. Each session lasted 30 min once per week. The program was carried out over 6 weeks, with a protocol minimum of at least 3 sessions. A mental health worker (either peer support worker, assistant psychologist, or clinical psychologist) was in the room when the therapy was provided. The staff had a wide range of clinical experience and were given half a day of training for VR therapy and weekly supervision. Their job was to help set up the system and provide guidance, but they did not actively participate in the program. The VR sessions were conducted in the participant's home or a clinic room. The usual care included antipsychotic medication, mental health worker visits, and psychiatrist appointments.

The main outcome was the Oxford Agoraphobic Avoidance Scale (OAS), measuring avoidance and distress in everyday situations. The secondary outcomes measured were agoraphobia (Agoraphobia Mobility Inventory-Avoidance scale), suicidal ideation (Columbia Suicide Severity Rating Scale), paranoia (Revised Green et al. Paranoid Thoughts Scale) and paranoia worries (Paranoia Worries Questionnaire), depression (Patient Health Questionnaire), and activity levels (measured using actigraphy over 7 days and a time budget assessing meaningful activity considering the complexity of activities and effort). Agoraphobic avoidance was also assessed using a behavioral assessment task (O-BAT) where the personalized hierarchy of five real-world situations was created and participants were asked to enter them in order of difficulty, stopping when unable to progress (O-BAT was originally the primary outcome, but changes to the study protocol were made due to COVID-19 pandemic). Ratings of distress were also obtained for each step completed. The study also measured quality of life (five-level EQ-5D, Recovering Quality of Life questionnaire, and Questionnaire about the Process of Recovery). Assessment of threat cognitions and use of within-situation defense behaviors as mediators were measured using the Oxford Cognitions and Defenses Questionnaire (O-CDQ) and strength of safety beliefs. Moderators were assessed at baseline with a short assessment of negative hallucinations when outside and through the Beck Hopelessness Scale, the Body Esteem Scale for Adolescents and Adults, and the O-CDQ. Medical notes were also checked after the trial for serious and not serious adverse effects.

The level of agoraphobic avoidance varied at baseline in the VR group (average in 17%, moderate in 32%, high in 29%, and severe in 22%, with data missing in 1%) and in the TAU group (average in 19%, moderate in 23%, high in 30%, and severe in 28%).

Compared to the TAU-only group, the VR-intervention group had a significant reduction in agoraphobic avoidance (*p* = 0.026, *d* = −0.18) and distress (*p* = 0·014, *d* = −0.26) at 6 weeks, as measured by the O-AS and large size reductions in O-BAT for agoraphobic avoidance and distress (in those who provided the data, *p* = 0.0004, *d* = 0.068 and *p* = 0.0.52, *d* = 0.43, respectively). The differences between the groups in O-AS scores were not statistically significant at 26 weeks.

The study found no significant differences in secondary outcomes between the two groups, except for improvement in the VR group in comparison to the control group in recovery assessed by a questionnaire about the process of recovery at 6 weeks and O-BAT avoidance at 6 and 26 weeks.

For avoidance and distress, the study found that threat cognitions and within-situation-defense behaviors (but not safety beliefs) significantly mediated treatment outcomes at 6 weeks, although at 26 weeks failed to reach statistical significance. Each of these mechanisms was found to separately explain approximately one-third of the treatment effect of the VR intervention.

Greater severity of threat cognitions (assessed by the O-CDQ) at baseline resulted in greater treatment benefits with the VR therapy at 6 weeks, indicating moderation by the severity of agoraphobia. The *post hoc* analysis of the primary outcome showed that the benefits of the VR intervention were only seen in patients with severe and high levels of agoraphobia, with the benefits maintained at 26 weeks.

Neither occurrence of negative verbal hallucinations, hopelessness, appearance concerns, age, or gender showed moderation effects.

### 5.4. Cognitive symptoms

A small study by La Paglia et al. (24) examined the effects of VR training in comparison to pharmacotherapy and integrated therapy in outpatients suffering from schizophrenia. In total, 15 patients were assigned to either VR training or control group without randomization (VR training, *n* = 9, control, *n* = 6). Both groups received normal psychopharmacologic treatment as well.

The VR cognitive training focusing on attention was delivered through the head-mounted device in three virtual environments with different, interactive tasks: a park, a valley, or a beach. The park environment trained sustained attention through catching balls shot at irregular intervals, the valley selective attention through identifying correct targets, and the beach both selective and divided attention, through identifying correct targets with interruptions. There was hierarchical sequencing for different tasks. The immersive VR cognitive rehabilitation intervention was implemented once a week over 10 individual sessions lasting ~90 min per session. The control group received 10 group sessions of IPT (integrated psychological therapy) once per week.

Before and after training, the study subjects were tested with neuropsychological assessments to overview general and executive cognitive functioning, sustained and divided attention, planning, brief and long-term memory, and cognitive flexibility through MMSE (Mini-Mental State Examination), FAB (Frontal Assessment Battery), TMT (Trail Making test forms A, B and B-A), ToL (Tower of London Test), Memory Battery, and WCST (Wisconsin Card Sorting Test) tests. The study also measured the following items at the first and last sessions of the VR intervention: task execution time and total errors made, requests for assistance and therapist's interventions, sustained and divided attention, maintained sequencing of the task, self-correction, absence of perseveration, and maintained task objective to completion.

In the study, both groups improved in performance on the divided attention task (TMT-B), with the VR training group showing better improvement in sustained attention (TMT-A, *p* = 0.033), general functioning (MMSE, *p* = 0.026), and planning (ToL, *p* = 0.042) when the improvement from baseline to the end of the intervention was measured, separately in each group. Over the VR training, the patients improved their execution times (*p* = 0.008) and had reduced need for assistance (lesser requests, *p* = 0.018 and therapist interventions, *p* = 0.008). They also reduced their errors (*p* = 0.042) and showed improvement in sustained attention (*p* = 0.046) in the tasks.

A study by Wang et al. (31) explored the potential benefits of adding cognitive training through immersive VR serious gaming to standard psychiatric care.

In total, 64 inpatients were randomized to either the experimental VR training group (*n* = 31) or the control group (*n* = 33). While the control group received only standard psychiatric care, the experimental group also played a VR game twice daily for 10 days.

The VR intervention component was ”Fruit Pioneer,” an active, serious VR game played from a first-person perspective, where the player (patient) tries to score points by cutting moving fruits and avoiding penalty-inducing iron balls. As the fruits could fly from any direction, the players had to stay vigilant of their surroundings in the virtual environment. As the game progressed, it became increasingly more demanding. Each game level was 2 min and there was a 10-second pause between each level. Each session lasted 20–30 min, twice daily. Each participant finished at least 20 sessions. A psychiatric nurse explained the game rules, recorded the sessions, and accompanied the patient in case of adverse effects. A diameter of 2 m was required as a safe space for the intervention. The control group received standard inpatient psychiatric care, such as medication and group psychiatric rehabilitation including psychoeducation about symptom and medication management, learning or occupational goal setting and plan making, and social skills training. The patients in the intervention group could also participate in these activities if they wanted to. The psychiatric treatment was based on medical needs.

The primary outcomes assessed were working memory, executive function, and verbal fluency associated with social cognition. These were tested through the Brief Cognitive Assessment tool for Schizophrenia (B-CATS), including the Digital Symbol Substitution Test (DSST), Trail Making Test parts A and B (TMTA and B), and Animal Fluency (AF) test.

No participant dropped out or failed to complete the training sessions. There was no difference in AF scores, but significantly higher scores for DSST (*p* = 0.001, *d* = 0.87) and faster completion times in TMTA (*p* = 0.023 d =0.59) and TMTB (*p* = 0.018 d =0.62) in the intervention group in comparison to the control group, indicating a therapeutic effect on executive functioning and working memory.

Vass et al. (30) explored the effects of an immersive Virtual Reality Theory of Mind-intervention (VR-ToMIS) in a population of 21 stable outpatients with schizophrenia in comparison to passive VR exposure in a pilot randomized controlled study. Three patients dropped out before the first VR session and one patient was excluded due to an adverse effect not related to the intervention, leaving ultimately 17 patients, who were randomized to either the VR intervention group (*n* = 9) or passive VR exposure control group (*n* = 8).

The structured intervention was based on cognitive and behavioral therapy principles and targeted the Theory of Mind (ToM); broadly defined as the ability to deduce the mental states of another person, which is impaired in patients suffering from schizophrenia. The intervention was carried out once a week over 9 weeks, with each session lasting 50 min. The program included one preliminary session and eight active sessions. Active sessions included 3 consecutive steps preceded by a short warm-up and reviewing of the activity between sessions (homework and self-monitoring for the intervention group) and key procedures for change (e.g., how to keep up conversations). The three steps were as follows: 1. Simulated social interaction with game avatars in immersive VR environments with structured and pre-recorded dialogue elements designed to induce ToM impairment through social cues (such as double-meaning sentences, overstatements, and irony) for later interventions; 2. After each simulation, the patient had to visualize the inferred emotions of the avatar by use of the Temporal Disc Controller (TDC) task, helping to differentiate inconsistencies between verbalized and visualized mental states for observation; 3. Finally, the experience of the simulation was reviewed with a psychotherapist, who used cognitive and metacognitive techniques to guide the patient to develop more adequate behavioral strategies. The learned strategies were then tested by repeating the prior simulation. The control group could freely explore virtual environments but could not interact with avatars and did not receive any intervention.

Patients completed baseline and post-treatment assessments for psychopathology (PANSS), neurocognitive skills (Repeated Battery for the Assessment of Neuropsychological Status, RBANS and Wisconsin Card Sorting Test, WCST-64), ToM (BCMET, faux pas test, and cartoon stories task were administered to test the ability of mental state attribution), pragmatic language skills (non-literal language processing through Hungarian metaphor and irony test, consisting of four subtests: metaphor, irony, implicatures, and semantic subtests), and quality of life (Lancashire Quality of Life Profile (LQoLP). After each VR session, the participants were assessed for symptoms of simulator sickness using the Simulator Sickness Questionnaire and after the last session, all participants in the VR-ToMIS group were asked for their subjective opinion on the intervention (5-point Likert Scale). One relative of each patient evaluated the perceived changes (5-point Likert Scale).

The study found significant improvements in the VR-intervention group on negative symptoms on PANSS scores in comparison to the control group [effect size was large, Cramer's phi (ϕ) = 0.58, *p* = 0.01], and statistically non-significant but small effect sizes for positive and affective symptoms and non-significant but medium effect sizes for cognitive symptoms and activity scores.

Regarding neurocognition tests, the only statistically significant improvements in the VR group in comparison to the control condition were seen in reduction of errors [WCST-64, number of correct responses, *p* = 0.05, effect size was large, Cohen's partial eta squared () = 0.24] and on visuospatial and attention subtests of RBANS with medium effect sizes (visuospatial *p* = 0.01, $\eta_{p}^{2}$ = 0.34, attention *p* = 0.02, $\eta_{p}^{2}$ = 0.32). On WCST-64 and RBANS, non-significant improvements with large effect sizes were also seen for rate-of non-perseverative errors and immediate memory subtests.

The study found significant between-groups differences favoring VR intervention in several other tests in comparison to the passive VR exposure, seen both for lower and higher order ToM, for cognitive and affective subcomponents, and underactive ToM-tests in cartoon test first- and third-order tasks (*p* = 0.04, $\eta_{p}^{2}$ = 0.24, *p* = 0.02, ϕ = 0.55, respectively), faux pas-test overall scores (*p* = 0.003, $\eta_{p}^{2}$ = 0.46), and faux pas empathy and detection scores (*p* = 0.01, $\eta_{p}^{2}$ = 0.37, *p* = 0.02, $\eta_{p}^{2}$ = 0.032 respectively), as well as in metaphor-irony subscore (*p* = 0.005, ϕ = 0.67).

VR intervention was also associated with significant changes regarding the understanding of inappropriateness in the faux pas test (*p* = 0.03, ϕ = 0.52) and faux pas detection (*p* = 0.007, $\eta_{p}^{2}$ = 0.40). Positive, but non-significant changes were seen for BCMET scores, second-order Tom (Cartoon stories), faux pas-overall, and empathy scores.

Regarding pragmatic language skills, a significant change between groups was seen only in the interpretation of metaphors (*p* = 0.03, ϕ = 0.50, large effect size).

No significant differences were found in quality of life. Patients found the intervention interesting, engaging, easy, and safe to use. Relatives (*n* = 7) who observed patients reported a reduction in distrust patients exhibited toward others and a positive change in being involved with and more willingness to initiate conversations.

### 5.5. Comorbid symptomology

In a small study, Fusco et al. (28) compared the effects of progressive muscle relaxation (PMR) training with or without augmentation through VR on learning the technique as a coping mechanism for stress and anxiety and the intervention's effects on anxiety (STAI-y, BAI) in a population of psychotic patients at medium intensity level psychiatric facility.

In total, 22 patients suffering from schizophrenia, or another psychotic disorder, were randomized into experimental and control groups (*n* = 11 in both groups). The intervention was divided into two phases. In phase one, both groups received two 45-min sessions on learning to recognize psycho-physical tension of situations perceived as threatening. In phase two, both groups received 2 months of progressive muscle relaxation training (PMR) with descending levels of intensity; First twice weekly for 2 weeks, then once weekly for 2 weeks, and then once every 2 weeks for a month. The experimental group (*n* = 11) learned the technique through an immersive VR scenario involving a beach overlooking an ocean, with guided voice and background music while the control group used a more conventional setting (*n* = 11). The relaxation technique was 10 min in duration in both groups. The measurements were carried out in Phase 1 and at the end of training (although the abstract mentions a follow-up at 6 months after the intervention, this was not in the results).

Both groups reported positively on subjective feelings of relaxation with the experimental group having less difficulty in stretching muscles. Reduction in anxiety symptomology was seen in both groups between baseline and end of training with VR-augmented PMR more effective in comparison (BAI, *p* < 0.006; STAI-Y, *p* < 0.005).

## 6. Adverse effects

In total, 8 out of 12 included studies reported measuring for adverse events during the study with 1 studying VR social training, 3 VR CBT-based interventions, and 3 VR avatar therapy-based interventions.

Park et al. (23) reported checking patients for subjective feelings of simulator sickness after each immersion with HMD in their study of VR social training. They reported no health problems related to the use of HMD, but their study-progress flowchart reports two dropouts in the VR group and one in the control group due to symptom aggravation.

Regarding the use of CBT-based VR therapies, Pot-Kolder et al. (26) reported no adverse events related to the VR-CBT but found discomfort, cybersickness, or fear related to the equipment use or therapy modality (*n* = 4). In total, 11 out of 58 (19%) patients in the intervention were dropped from the study; either never starting the intervention (*n* = 4) or discontinuing due to logistics (*n* = 2), equipment-related discomfort (nausea, *n* = 1, HMD too uncomfortable *n* = 2), being afraid to continue (*n* = 1), or not being able to comply alcohol sobriety (*n* = 1). Vass et al. (30) report that no patient dropped out during VR intervention (ToMIS). One patient was excluded due to an adverse event, which was not related to the intervention and 3 patients did not start the intervention. Although the study reports assessing symptoms of simulator sickness with SSQ after each VR use, it does not report results. Cella et al. (24) report 2 serious adverse events and four adverse events in the control group and seven adverse events in the VR intervention group during the study period. None of the events were considered to be associated with study participation. Freeman et al. (11) studying automated VR-CBT reported 25 adverse events from 21 different patients (12 were serious in nine patients) in the intervention group. In the TAU group, there were 29 adverse events from 19 different patients (eight in seven patients were considered serious). In addition, 10 out of 12 serious adverse events in the VR group were considered to be “definitely not related” to the intervention and 2 out of 12 were “probably not related.”

All the studies utilizing VR-based avatar therapy; either VRT or CATS, mentioned measuring for adverse events. In the study by du Sert et al. (27), 4 out of 19 patients dropped out due to anxiety or lack of engagement in intervention after the first VRT session. The study reports the first 2 weeks as most anxiogenic, with significant reductions in anxiety and fear seen at week 4. No rehospitalizations occurred during the study, but one patient entered a counseling and support center temporarily at week 1 of VRT. Dellazizzo et al. (29) report in their 1-year follow-up trial studying VRT in comparison to CBT, that 12/74 withdrew from the study intervention groups (9 from VRT and 3 from CBT), with differing reasons (lack of motivation, not wanting to reduce voices, and moving away). The study reports no rehospitalizations during the totality of the trial. Moreover, 37.5% of the patients in the VRT group found the therapy stressful in the beginning, but after that, found it interesting and enjoyable. Liang et al. (29) report that there were no adverse events related to either CATS or the CBT-control condition but does not specify how the adverse events were screened for.

## 7. Requirements for VR interventions

### 7.1. Technical requirements

We have gathered the technological information available from the study documents in Table 3 below.

**Table 3:** Technological requirements for VR intervention.

**Study**	**HMD**	**Other technology required**	**Software used**
Park et al. (23)	Eye Trek 250W, Olympus	Joystick, Computer (rendering and virtual environment), 120-inch screen where others in the group can follow what happens inside an immersive environment., Position tracker (InterTrax2, InterSense) for following head direction.	Not mentioned
La Paglia et al. (24)	Non-specified HMD	Non-specified trackers and a joypad, computer for accessing VR-environment	Neuro-VR 2.0 software for accessing VR environment.
Freeman et al. (25)	nVisor SX111	12 Interse Sonistrip ceiling and Intersense IS-900 SimTracker system to specify for viewer's position and orientation. Computer for running VR application (custom build; core i7 processor, NVIDIA GeForce GTX 780 ti graphics card with 3072 mb of memory. 16GB of RAM. Asus Maximus VII Ranger motherboard. Computer for tracking (Dell T5500 workstation with a core i7 processor and 4 GB RAM. Audio rendering with Realtek audio controller.	Train environment rendering: XVR application platform. Lift environment rendering: Unity3D application platform.
Pot-Kolder et al. (26)	Sony HMZ-T1/T2/T3	Logitech F310 Gamepad for movement in VR environment. 3DOF tracker for head rotation.	Vizard Software
Du Sert et al. (27)	Samsung GearVR	Samsung Galaxy S6 smartphone for running software.	AVATAR creation: Idiosyncratic avatars: Unity 3D game engine and Morph3D Character System. The avatar's voice simulation with Roland AIRA VT-3 voice transformer, lip synchronization prosody of intonation and language through SALSA via RandomEyes Unity 3D extension
Fusco et al. (28)	Non-Specified HMD	Headphones for relaxation instructions	Not mentioned
Dellazizzo et al. (29)	Samsung GearVR or Oculus Rift	Not mentioned.	AVATAR creation: Idiosyncratic avatars: Unity 3D game engine and Morph3D Character System. The avatar's voice simulation with Roland AIRA VT-3 voice transformer, lip synchronization prosody of intonation and language through SALSA via RandomEyes Unity 3D extension
Vass et al. (30)	Samsung Gear VR	A Samsung S7 smartphone for running the software and a Samsung simple controller for interaction in the VR environment.	VR environment by vTIme
Wang et al. (31)	HTC Vive	Computer for running software: HP PC, Intel Core i5-9400F processor, 16 GB DDR4 3000 MHz memory, GTX 1660 6GB graphics card, 256 GB solid-state drive, and 1 TB hard disk drive. Handle-held devices were used for interaction. HMD-linked headphones were used for audio.	Software developed using Unity3D
Freeman et al. (11)	HTC Vive Pro	Computer: Dell G5 15 5590.	Software programmed by Oxford VR.
Cella et al. (32)	Oculus Rift-S	VR-ready laptop to run the software, ear-covering headphones for sound.	VR environments were designed by Virtualware (Unity).
Liang et al. (33)	HTC Vive	MP3-player	Intervention: CATS software. AVATAR creation: 2D creation of avatar and generation to 3D with headshot plug-in in Character Creator (Reallusion, part of CATS software) and then creation of idiosyncratic character through Unity 3D Game engine and Blendshape. Voice simulation through voice conversion technology (Faceware Live Server) and facial and full-body motion capture (Perception Neuron PRO).

### 7.2. Human resource requirements

We have gathered the human resources requirements information available from study documents in Table 4 below.

**Table 4:** Human resources requirements for VR interventions.

**Study**	**Human resources required for intervention**	**Manualized**	**Individual or group sessions**	**Length of session (minutes)**	**Intensity of treatment**	**Number of sessions**	**Body position of patient during intervention**
Park et al. (23)	Main therapist (education level not specified) and co-therapists (social workers).	Yes	Group (size 4-5)	90	semiweekly	10	Not mentioned
La Paglia et al. (24)	Not mentioned	Yes	Individual	90	Weekly	10	Not mentioned
Freeman et al. (25)	A clinical psychologist explained experimental conditions and a research worker conducted assessments.	Not mentioned	Individual	60-90 (30 min in VR)	Once	1	Standing
Pot-Kolder et al. (26)	VR CBT therapists (Psychologists with at least basic CBT training) received two days of training in VR-CBT.	Yes	Individual	60 (40 min VR)	16 sessions over 8-12 weeks	16	
Du Sert et al. (27)	Therapy was delivered by a psychiatrist with 5 years of experience, with clinical experience of the sample population. The therapist animated the avatar's dialogue.	Yes	Individual	45	Weekly	1 avatar creation session and 6 therapy sessions	Not mentioned
Fusco et al. (28)	Not mentioned	Not mentioned	Not mentioned	Psychoeducation session: 45 min, VR: relaxation: 10 min	Phase I: 2 psychoeducation sessions (Non-VR), Phase II: VR sessions semiweekly for 2 weeks, then weekly for 2 weeks, then every 2 weeks for a month.	Phase I: 2 Phase II: 8	Not mentioned
Dellazizzo et al. (29)	Therapy was delivered by an experienced psychiatrist with 7 years of experience, with clinical experience of the sample population. Patients sat in an adjacent separate room from the therapist who animated the voice of the avatar or spoke as themselves, keeping up a trialogue.	Yes	Individual	60 (Obtained from study pre-registration)	Weekly	1 avatar creation session and 6 to 8 therapy sessions	Not mentioned
Vass et al. (30)	A trained psychotherapist delivered the intervention.	Yes	Individual	50	Weekly	9	Not mentioned
Wang et al. (31)	A psychiatric nurse explained the game rules and accompanied the patient in case of adverse events.	No	Individual	20–30	Twice a day	Min. 20	Standing
Freeman et al. (11)	1 mental health worker (Peer support workers, assistant psychologists, or clinical psychologists with a wide range of clinical experience and half a day of training for VR modality) was in the room to help set up and explain the procedure. The therapy was guided by an in-game virtual coach.	Yes	Individual	30	Weekly	6	Not mentioned
Cella et al. (32)	Therapist (Therapists were graduate-level psychologists with clinical experience with the target population)	Yes	Individual	Not mentioned	Not mentioned	12	Sitting
Liang et al. (33)	CATS team (psychiatrists and therapists with ~5 years of experience.). Therapist in an adjacent room alternating as a therapist or animating and voicing the AVH in a trialogue.	Yes	Individual	60	Weekly	1 avatar creation session and 6–8 therapy sessions	Not mentioned

## 8. Discussion

In our review, we found 12 studies using immersive VR and no studies using AR in the treatment of patients suffering from schizophrenia spectrum disorders.

There are several possible reasons why the general academic interest is focused more on VR solutions. First, and most important, the studied therapeutic modalities utilize the unique possibilities provided by immersive VR; such as the use of virtual environments and intersocial interactions with precoded game characters (avatars) for safe, easily repeatable, and otherwise demanding treatment modalities (e.g., exposure-based methods). AR solutions either lack the immersivity (depending on the chosen media) offered through VR-HMD or the capability to meaningfully alter virtual surroundings. The second important factor is the level of commercial availability; head-mounted devices for immersive VR have become more affordable and thus commercially available while there are less easy-to-use immersive AR solutions fitting for psychiatric interventions. It is possible that commercially available non-immersive AR technology still lacks the aforementioned qualities needed to augment therapy modalities. It could be argued that modalities that focus on communication with a single avatar and do not focus otherwise on surroundings (such as avatar therapy-based modalities) could utilize AR-based methods. For example, one study targeting stigma toward schizophrenia patients already demonstrated the possible use of augmenting physical reality with pseudohallucinations (34). There is no available evidence as of yet to argue whether such methods would or would not augment avatar therapy. Overall, there is a research gap regarding the use of AR in the treatment of schizophrenia.

The studied VR-based intervention modalities were diverse in nature, with most studies using VR to augment CBT-based techniques (*n* = 5). Other modalities are either augmented avatar therapy (*n* = 3), otherwise utilized virtual environment (*n* = 2), or gamified treatment (*n* = 2). These modalities had most likely been chosen either as they were based on previously proven psychological treatments (social roleplay, CBT, and avatar therapy) or due to the ease of creating a study setting (relaxation and serious gaming for neurocognitive training). While receiving VR interventions, the patients also received their standard psychiatric treatment.

VR seems a useful tool to utilize in CBT because it allows for a safe method to study the behavior in-virtuo; a gradual, less intense way than real life to habituate the patient into real-life situations, while also allowing the therapist to observe, support, and correct the patient's behaviors in real time, as well as allowing for easy replication of prior situations so they can be gone over multiple times for reinforced learning. The range of CBT-based modalities was diverse with different targets such as lowering safety behaviors to reduce paranoid ideation, social distress, and agoraphobia, improving social functioning, and ameliorating negative or ToM deficits as well as motivational deficiencies. In the included studies, VR-CBT was used to augment treatment as usual in comparison to either TAU-alone or simple VR exposure and found positive effects on all priorly mentioned targets. Freeman et al. (21) found a single session of VR-CBT used to reduce safety behaviors in the virtual environment to reduce paranoid ideation and distress related to paranoia in VR settings and subsequently to reduce real-life social distress in comparison to VR exposure without the CBT component. Pot-Kolder et al. (25) also reported that 16-session VR-based CBT in comparison to wait-list control (TAU), reduced momentary paranoid ideation, momentary anxiety regarding social situations, and use of safety behaviors, with the effects maintained at 6-month follow-up. The treatment on the other hand did not increase social participation. Cella et al. (32) found positive changes in goal attainment of preselected goals through their 12-session VR-CBT modality (V-Nest) and Vass et al. (30) showed that a 9-session VR-CBT targeting ToM deficits improved negative symptomology (PANSS score), ToM deficits, and neurocognition in comparison to simple VR exposure. Freeman et al. (11) also exhibited that a self-guided, ambulatory VR-CBT modality (GameChange) in comparison to wait-list control (TAU) over 6 weeks and with a minimum of 3 sessions reduced agoraphobic avoidance and distress at post-treatment, although at follow-up at 26 weeks failed to reach statistical significance, except for those rated highly or severely agoraphobic. Overall, the studies show preliminary evidence supporting the use of VR-based CBT interventions but require larger studies to validate their findings. VR-CBT also needs to be compared with other, priorly validated treatment modalities (such as regular CBT) to see whether augmentation of treatment using VR has further clinical significance.

VR social training, by using precoded avatars in the virtual environment to help patients train their social skills, is closely related to CBT techniques of exposure and holds the same benefits offered by the virtual environment. Park et al. (23) in their 10-session study showed that such a method in comparison to traditional social roleplay (through verbal, picture, video material, and acting) had an advantage when it came to learning conversational skills, but a disadvantage in learning non-verbal skills. This is understandable, since as of 2011 (and possibly as of now as well), when the study was published, VR graphics were not up to a point, that could properly and realistically enough animate non-verbal cues.

Avatar therapy has priorly been studied through computerized methods and its effect on patients with schizophrenia is still uncertain (35). In the studies included, avatar therapy using immersive VR had an ameliorating effect on persecutory hallucinations in patients with treatment-resistant schizophrenia. The studies show that augmenting trialogue among patient, virtualized avatar of the AVH, and therapist by adding 3-dimensionality and life-likeness through VR and lip-sync, voice transformation (27, 29), and even body motion capture technology (33) for movement to add a higher level of immersivity and realism to the therapeutic experience could be of clinical significance, although we still require more research on the subject; especially comparing screen-based and VR-augmented methods.

For VR training, two studies showed that gamification of treatment through neurocognition-enhancing tasks (24, 31) in VR could be used to improve neurocognition. The first study by La Paglia et al. (24) compared 10-session VR training in different settings with different tasks to integrated psychological treatment. It found that both groups improved scores on divided attention and VR training on general functioning and planning. The patients in the VR group also improved their scores on the training tasks that they were doing. Wang et al. (31) compared the 20-session VR training to treatment as usual. An active VR serious game “Fruit pioneer” improved scores on executive functioning and working memory in comparison to treatment as usual.

One study by Fusco et al. (28) showed that including a relaxing VR environment for progressive muscle relaxation could help patients learn the technique better for stress relief and thus reduce anxiety.

The included studies found close to no adverse effects related to the VR intervention modalities; indicating a safe profile for VR use. Even intense treatment such as immersive avatar therapy led to no adverse effects in the study settings in the long run, with the introductory and starting period of the treatment being the most anxiogenic, in line with general effects related to exposure treatments (27, 29). Most of the found adverse effects mediated by the treatment were mild and related to the use of HMD, such as simulator sickness. This adverse effect is likely to lessen in future as technological solutions evolve. It is important to note that patients suffering from schizophrenia seem to rely more on sight than other mechanisms for balance and postural stability and whether the inability to rely on sight during the VR intervention could lead to more falls in the patient group (36). Thus, far we have also yet to find whether there are any clear contraindications for the use of VR other than physical incapability to use the equipment. Although the exclusion criteria of the studies might give some guidance regarding this, more research is required to find out which patient characteristics increase potential adverse effects. The ethical questions related to the use of emerging technology also require further research (37).

Regarding the use of VR in general, the HMDs and adjacent technology are getting cheaper with more commercial availability. Most of the attached costs of the treatments are likely to come from research and development costs of virtual treatment modalities, as well as future licensing prices for treatments and from the human resources required to conduct the treatment. In most cases, the augmentation of CBT and avatar therapy through VR still requires a highly trained professional to deliver the intervention and guide it. Freeman et al. (11) have offered an interesting solution to have a pre-programmed in-game guide to deliver the intervention modality. This could allow for mass production of such interventions, freeing mental health professionals from other duties, and possibly giving patients more responsibility and freedom in their treatment.

The included studies were diverse in the quality of their reporting as well as regarding their risks for bias (see Figures 2, 3). The studies were mostly of good quality, with the bias exhibited mostly unavoidable in the study setting, except for two studies by La Paglia et al. (24) and Fusco et al. (28) with scarce reporting and lack of informing data. Due to a lack of information, the study by La Paglia et al. (24) could not be properly assessed. The study by Fusco was flagged for high risk for bias. Caution should be exercised when attempting to replicate the results. The study by du Sert et al. (27) although well-reported also carries a high risk for bias because of the intervention leading to a significant amount of drop-out due to anxiety and non-inclusion of dropouts in the analyses and the cross-over design. It is important to note that multiple studies were exploratory in nature (feasibility and pilot trials). Regarding the assessed potential risks for bias in general, when delivering an intervention through a psychosocial method and a distinct device such as HMD, the patient, and assessor cannot understandably be fully blinded to the intervention received. Some of the studies tried to answer this challenge by comparing the active VR intervention to a passive VR environment or another active psychosocial intervention (such as CBT). The use of patient-reported outcomes of symptoms, especially without blinding the participants to the intervention, can introduce bias in such situations. Also, in some of the VR studies, the patients either really enjoyed the intervention or felt the intervention anxiety inducing; although this is a potential part of the treatment effect, it might lead to a difference in adherence rate, and if not accounted for, could lead to bias in the study.

Multiple studies which were excluded were either non-interventional studies of assessment and evaluation, feasibility studies not studying intervention effects, without a comparison group or included participants with mood disorder-based psychosis or healthy population, and included non-immersive methods or did not target pre-specified target symptoms (e.g., studies of public stigma reduction). These studies could be of interest to clinicians and researchers but were out of the scope of this review.

In conclusion, VR solutions allow for a completely new way of treatment in comparison to standard psychopharmacologic and psychosocial treatments to affect patient behavior and ameliorate multiple domains of their symptoms with so far, few identifiable problems related to the treatment. The gamification of treatment has the potential to inspire the patients to better engage in their own treatment, although this might lessen if the novelty felt toward the technology wears off in the future. With especially pharmacologic treatment carrying with it significant side-effect burden, new treatment modalities to augment the regular treatment are most likely to be welcomed by the patients and their close ones to motivate and help them better adhere to their treatment regimen. This line of thinking advocates for further research into VR as a treatment vector for interventions targeting patients suffering from schizophrenia spectrum disorders. Regarding AR, there is no research on immersive treatment interventions available targeting this patient group, indicating a clear research gap.

## Author contributions

ML and RH planned the study protocol. RH conducted the literature review, analysis of studies, and wrote the initial draft of the manuscript. The risk-of-bias assessment was done by RH and ML. ML had a supervising role in the study. ML and JT revised the manuscript for important intellectual content. All authors contributed to the article and approved the submitted version.
